# Praziquantel Synergistically Enhances Paclitaxel Efficacy to Inhibit Cancer Cell Growth

**DOI:** 10.1371/journal.pone.0051721

**Published:** 2012-12-12

**Authors:** Zhen Hua Wu, Ming-ke Lu, Long Yu Hu, Xiaotong Li

**Affiliations:** State Key Laboratory of Cellular Stress Biology, School of Life Sciences, Xiamen University, Xiamen, Fujian, P.R. China; Bauer Research Foundation, United States of America

## Abstract

The major challenges we are facing in cancer therapy with paclitaxel (PTX) are the drug resistance and severe side effects. Massive efforts have been made to overcome these clinical challenges by combining PTX with other drugs. In this study, we reported the first preclinical data that praziquantel (PZQ), an anti-parasite agent, could greatly enhance the anticancer efficacy of PTX in various cancer cell lines, including PTX-resistant cell lines. Based on the combination index value, we demonstrated that PZQ synergistically enhanced PTX-induced cell growth inhibition. The co-treatment of PZQ and PTX also induced significant mitotic arrest and activated the apoptotic cascade. Moreover, PZQ combined with PTX resulted in a more pronounced inhibition of tumor growth compared with either drug alone in a mouse xenograft model. We tried to investigate the possible mechanisms of this synergistic efficacy induced by PZQ and PTX, and we found that the co-treatment of the two drugs could markedly decrease expression of X-linked inhibitor of apoptosis protein (XIAP), an anti-apoptotic protein. Our data further demonstrated that down-regulation of XIAP was required for the synergistic interaction between PZQ and PTX. Together, this study suggested that the combination of PZQ and PTX may represent a novel and effective anticancer strategy for optimizing PTX therapy.

## Introduction

It became a new trend that turning an old drug for new uses especially for cancer treatment, because those routinely used old drugs might have a hidden talent or good potential in dealing with cancer. The fact is that all workup has been done already, which allows us to move the drug into the clinical more quickly and to reduce the cost for drug development [1], [2]. The concept of “new uses for old drug” provides an efficient way to rediscover new uses for existing drugs with known pharmacokinetics and safety profiles. Some successful examples for this type of cancer drug development were previously reported such as Thalidomide [3], Vitamin C [4]–[6], NSAIDs (Nonsteroidal anti-inflammatory drugs) [7]–[11]. Recently, it has been reported that Artemisinin, an anti-parasite agent, and its derivatives, had profound cytotoxicity against cancer cells from different tumors [12]–[16], providing the impetus to develop anti-parasite drugs into anticancer drugs. Praziquantel (PZQ), another anti-parasite agent, has been widely used to treat different schistosomiasis with good efficacy [17], [18]. Interestingly, it was reported that PZQ can enhance the humoral and cellular immune responses of the host against diseases [19], [20]. It would be interesting to investigate whether PZQ has anticancer activity which is still unclear so far.

In this study, killing activity of PZQ on cancer cells was assessed with different assays. We also investigated the effects of combined treatment with PZQ and the commonly used chemotherapeutic drug paclitaxel (PTX). PTX is a microtubule-stabilizing agent which can promote microtubule stabilization, resulting in the arrest of cells in G2/M phase of cell cycle and leading to apoptosis [21], [22]. As one of the most commonly used anticancer drugs, PTX has demonstrated strong efficacy against a wide range of malignancies, including breast, head and neck, ovarian and non-small cell lung cancers, as well as Kaposi’s sarcoma [23]. However, emergence of clinical resistance and broad range of severe side effects remain significant problems with PTX therapy [24]–[26]. Consequently, numerous recent studies focused on the PTX synergistic therapy aiming to find an effective solution for overcoming PTX-resistant problem and reducing toxicity induced by PTX without compromising the drug efficacy [27], [28].

Here, we reported that PZQ could synergistically enhance the growth-inhibitory effect of PTX in a variety of cancer cell lines, including PTX-resistant cell lines such as DLD1 and H1299, although PZQ treatment alone did not exert cytotoxicity on these cancer cells. PZQ could also greatly enhance PTX-induced mitotic arrest and apoptosis. In further studies, we showed that this cytotoxic synergy between PZQ and PTX involved down-regulation of XIAP. The ability of PZQ to potentiate the anticancer effects of PTX was subsequently confirmed in a mouse xenograft model. These results provided important implications for optimizing PTX therapy. Combining PZQ with PTX may represent a novel and effective anticancer strategy.

## Materials and Methods

### Cell Lines and Cell Culture

Human colon cancer cell line DLD-1, breast cancer cell line ZR-7530, lung cancer cell lines SPC-A-1 and Ltep-a-2 were cultured in RPMI 1640. Human non-small-cell lung cancer cell line H1299, cervical cancer cell line HeLa and human breast cancer cell line Bcap37 were maintained in DMEM. All media were supplemented with 10% (v/v) fetal bovine serum (GIBCO, Carlsbad, CA), 100 units/mL penicillin and 100 mg/mL streptomycin. Cells were maintained at 37°C in a humidified atmosphere of 5% CO_2_. All cell lines were obtained from the Cell Bank of the Chinese Academy of Sciences (Shanghai, China). Cell lines were free of mycoplasma when tested by a PCR-based mycoplasma test [29], [30].

### Reagents and Antibodies

Paclitaxel (PTX), roscovitine, the rabbit polyclonal antibody against Bim, Puma, and the mouse monoclonal antibody (mAb) against β-actin were obtained from Sigma-Aldrich (St. Louis, MO). Praziquantel (PZQ) was kindly provided by Dr. Jun Lu (Nanjing Pharmaceutical factory co., LTD, Nanjing, China). MG132 was from Calbiochem (Darmstadt, Germany). The rabbit polyclonal antibody against cleaved poly (ADP-ribose) polymerase (PARP; p89) and phospho-histone H3 (Ser-10) (P-H3) were purchased from Cell Signaling Technology (Beverly, MA). The rabbit polyclonal antibody against Noxa, bax, bak, caspase-3, Survivin, Bcl-2, and Bcl-X_L_ were from Santa Cruz (Santa Cruz, CA). The mouse mAb against XIAP was from BD Transduction Laboratories (San Diego, CA). Goat anti–rabbit and goat anti–mouse horseradish peroxidase–conjugated secondary antibodies were purchased from Pierce Biotechnology (Rockford, IL).

### Cell Viability Assay

Cell viability was determined by 3-(4, 5-dimethylthiazol-2-yl)-2, 5-diphenyltetrazolium bromide (MTT) assay. Cells plated in 96-well plates were incubated with indicated drugs at 37°C for different periods of time. Then the culture medium was removed and medium containing MTT (0.5 mg/ml) was added to each well. The plates were incubated for an additional 2–4 h in a CO_2_ incubator at 37°C. The formazan crystals were dissolved in 100% DMSO and absorbance at 570 nm was measured using a microplate reader. Every sample was measured in triplicate and repeated three times. The relative percentage of survival was calculated by dividing the absorbance of treated cells by that of the control in each experiment. PTX and PZQ as single agents and in combination at the highest concentrations used in the reported experiments do not interfere with the MTT assay reagents in our control experiments (data not shown).

### Cell CycleAnalysis

For analysis of DNA content and cell cycle by flow cytometry, cells were harvested, washed once with PBS, and fixed in 70% ethanol at 4°C over night. At the time for flow cytometry analysis, cells were washed with PBS and re-suspended in staining solution (100 µg/mL RNase and 100 µg/mL propidium iodide in PBS). After cells were incubated for 30 min at 37°C in the dark, the cell cycle distribution was analyzed by a Coulter EPICS XL flow cytometer (Beckman Coulter, Miami, FL).

### Western Blot Analysis

Western blot analysis was performed as described previously [31]. Briefly, cells were harvested and lysed in lysis buffer containing 20 mM Tris-HCl (PH = 7.4), 150 mM NaCl, 1% Triton-X 100, 1 mM phenylmethanesulfonyl fluoride, 10 µg/mL leupeptin, 2 µg/mL aprotinin, 10 mM NaF, 1 mM Na_3_VO_4_, and the protein concentration was determined by using the bicinchoninic acid (BCA) assay. Cellular proteins were subjected to sodium dodecyl sulfate-polyacrylamide gel electrophoresis and transferred to the polyvinylidene difluoride membrane (Millipore, Bedford, MA). Membranes were blocked with TBS-Tween 20 (0.5%) containing 5% nonfat milk for 2 h at room temperature and incubated with primary antibodies overnight at 4°C. After three washes with TBS-Tween 20, membranes were incubated with goat anti–rabbit or goat anti–mouse horseradish peroxidase–conjugated secondary antibodies for 1 h at room temperature. The membranes were washed with TBS Tween-20 again and developed by enhanced chemiluminescence (Pierce, Rockford, IL). β-actin was used as a loading control. The primary antibodies were used at a 1∶1000 dilution, except for anti-β-actin, which was used at a 1∶5000 dilution. The anti–rabbit or anti–mouse horseradish peroxidase–conjugated secondary antibodies were used at a dilution of 1∶5000.

### DAPI Staining

After drug treatment, cells grown in 6-well plates were washed with PBS and fixed with 3.7% formaldehyde for 10 minutes at room temperature, then incubated with the staining solution (0.3% Triton-X 100 and 1 µg/mL DAPI in PBS) for 5 minutes avoiding light at room temperature. Cells were examined by fluorescence microscopy (Nikon). Cells having fragmented and uniformly condensed nuclei were regarded as apoptotic cells, and apoptosis was expressed as a percentage calculated from the number of cells with apoptotic nuclear morphology divided by the total number of cells examined.

### Colony Formation Assay

Cells were seeded into 6-well plates at 1.5×10^3^ cells per well and exposed to drug treatment. After 10 days, the cells were fixed and stained with crystal violet (0.5% crystal violet and 20% methanol) for 30 minutes. Then the plates were washed with distilled water and photographed. Colonies that contained more than 50 cells were counted. Values for each condition were expressed as a percentage relative to vehicle-treated controls. Each assay condition was performed in at least three independent experiments.

### Immunofluorescence Staining

Cells on coverslips were fixed in 4% paraformaldehyde in PBS at room temperature for 10 min, washed with PBS, permeabilized with 0.2% Triton-X100 in PBS for 10 min, and then blocked with 3% bovine serum albumin in PBS at room temperature for 30 min. Anti-Phospho-Histone H3 (Ser-10) (P-H3) antibody (dilution 1∶100) was added and incubated for 30 min at room temperature. After washing with PBS containing 0.02% Triton X-100 and 1.5% BSA, the coverslips were incubated with Alexa Fluor 647-conjugated goat anti-rabbit IgG (dilution 1∶200) for 30 min at room temperature. Finally, the cells were incubated with 1 µg/ml DAPI in PBS for 5 minutes before mounted in 90% glycerol and sealed with nail polish. Mitotic index was expressed as a percentage of P-H3-positive-cells relative to the total number of cells examined.

### Plasmids, Cell Transfection and RNA Interference

The XIAP-expressing plasmid pcDNA3-myc-XIAP was a gift from Dr. John C. Reed (The Burnham Institute, La Jolla, USA) and the empty pcDNA3 was used as a negative control. Cell transfection was performed using Lipofectamine 2000 (Invitrogen, Carlsbad, CA) according to the manufacturer’s instructions.

To knockdown XIAP, shRNA targeting XIAP sequence (GTGGTAGTCCTGTTTCAGC) was cloned into a lentiviral vector Lenti-Lox3.7 (pLL3.7). A sequence with no corresponding part in the human genome (GATCATGTAGATACGCTCA) was used as a control. For production of infectious shRNA–expressing lentiviruses, 293 T cells were transfected with pLL3.7 lentiviral vector together with packaging vectors pVSV-G, pRSV-Rev and pMDL gag/pol RRE. 48 h post-transfection, the virus-containing supernatant media were harvested, passed through a 0.45-µm filter and used to infected cells after addition of 10 µg/mL Polybrene.

### Real-time Reverse Transcription-PCR

Total RNA was extracted using TRIzol reagent (Invitrogen, Carlsbad, CA) according to the manufacturer’s instructions. First-strand cDNA was synthesized from the total RNA using an oligo-dT primer and Moloney murine leukemia virus reverse transcriptase (TaKaRa, Shiga, Japan). Real-time PCR was performed on the Rotor-Gene 6000 (Corbett Research, Mortlake, Australia) and SYBR green (BIO-V, Xiamen, China) was used as a detection reagent. The primer sequences for XIAP and glyceraldehyde-3-phosphate dehydrogenase (GAPDH) were: XIAP (forward: 5′-GACAGTATGCAAGATGAGTCAAGTCA-3′; reverse: 5′-GCAAAGCTTCTCCTCTTGCAG-3′), GAPDH (forward: 5′-CCACCCATGGCAAATTCC-3′; reverse: 5′-TGGGATTTCCATTGATGACAAG-3′). Each sample was run in triplicate. Data analysis was performed with the Rotor-Gene 6000 series software 1.7 (Corbett Research, Mortlake, Australia). The relative RNA amounts were normalized to GAPDH mRNA.

### Xenograft and Treatment Procedures

All animal procedures were approved by the Animal Care and Use Committee of Xiamen University. Tumor cells (DLD-1, 2×10^6^) were injected subcutaneously into athymic nude mice (BALB/c, 4–5 weeks old). When tumor volume reached 25–35 mm^3^, animals were randomized and assigned to different treatment groups (n = 6 in each group). Animals were injected intraperitoneally with PZQ alone (100 mg/kg), PTX alone (30 mg/kg), or a combination of PZQ (100 mg/kg) and PTX (30 mg/kg). PTX was dissolved in equal volumes of Cremophor EL and ethanol, and further diluted with PBS before injection. PTX was given on days 5, 9, and 13 after tumor cell injection. PZQ dissolved in corn oil was given on days 5, 7, 9, 11, and 13 after tumor cell injection. For single-agent treatment, vehicle was given in place of PZQ or PTX with the same schedule. Tumor size was determined by using calipers. Tumor volume (mm^3^) was calculated by the formula: (a) × (b^2^) × 0.5, where a is tumor length and b is tumor width in millimeter.

### Statistical Analysis

Values were expressed as mean±SEM. To determine significant differences in the data, the two-tailed unpaired Student’s *t* test was applied. Differences were considered to be statistically significant at P<0.05. Drug interactions were examined for synergism or antagonism using Median Dose Effect analysis (Calcusyn; Biosoft, Ferguson, MO).

## Results

### PZQ does not Exert any Cytotoxicity on Tumor Cells

The cytotoxicity of PZQ on various tumor cells was first examined with tumor cell lines including lung cancer cells (SPC-A-1, Ltep-a-2 and H1299), breast cancer cells (Bcap37 and ZR7530), cervical carcinoma cells (HeLa) and colon cancer cells (DLD-1). However, PZQ induced neither growth inhibition nor apoptosis even at concentrations as high as 100 µM (Fig. S1), indicating that PZQ did not exert any cytotoxicity on tumor cells.

### The Co-treatment of PZQ and PTX Synergistically Inhibits Tumor Cell Growth

**Figure 1:**
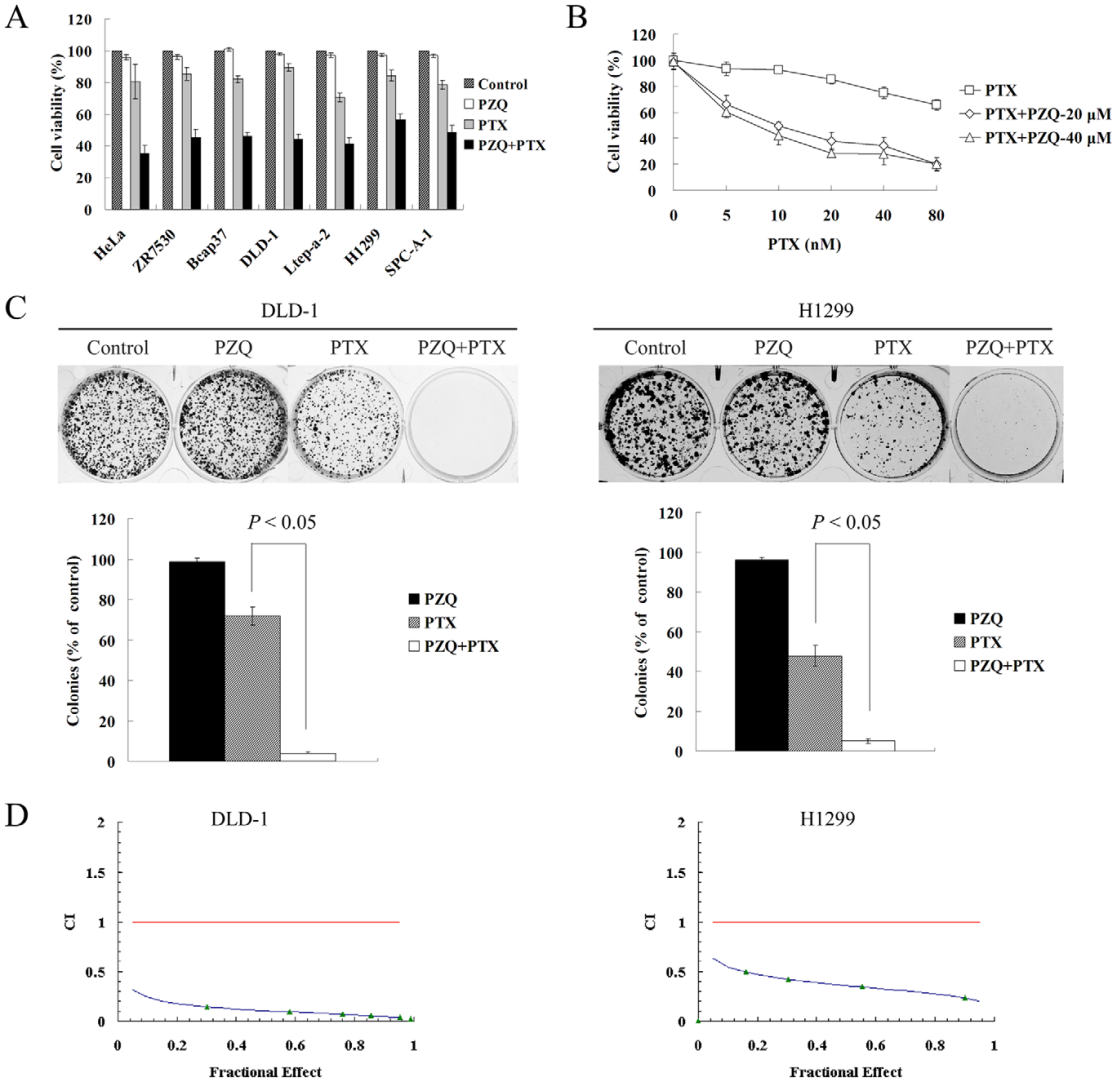
PZQ synergistically enhances PTX-induced growth inhibition of cancer cells. (A) The cell viability was determined by MTT assay after various cancer cell lines were treated with PZQ alone, PTX alone or combined both drugs at concentrations and time points as indicated following: HeLa, ZR7530, DLD-1 and H1299 cells were treated with 20 µM PZQ alone, 10 nM PTX alone, or combined both for 48 h; Bcap37 and SPC-A-1 cells were treated with 30 µM PZQ, 5 nM PTX alone, or both for 48 h; Ltep-a-2 cells were treated with 30 µM PZQ alone, 5 nM PTX alone, or both for 60 h. (B) DLD-1 were treated with the indicated concentrations of PTX in the absence or presence of 20 or 40 µM PZQ for 48 h. Cell viability was then determined by MTT assay. (C) DLD-1 and H1299 cells were treated with 20 µM PZQ alone, 10 nM PTX alone, or the combination for 10 days. Colonies were then stained with crystal violet and counted. (D) DLD-1 and H1299 cells were treated with various concentrations of PZQ and PTX at a fixed ratio (2000∶1) for 48 h. After cell viability was determined in each condition, the combination index (CI) was calculated as described in “Materials and Methods.” CI values<1.0 suggest a synergistic interaction between the two drugs. All Values represent mean±SEM from three separate experiments.

We next evaluated whether PZQ could affect sensitivity of tumor cells to chemotherapeutic agents such as paclitaxel (PTX) by MTT assay. We found that PZQ greatly enhanced the growth inhibition by PTX in various tumor cell lines including two PTX-resistant cell lines, DLD-1 and H1299 (Fig. 1A, Fig. S2). We did most of our following experiments with these two PTX-resistant cell lines. To gain further insight into this combination effect, dose-response studies were performed as showed in Fig. 1B. PZQ could enhance sensitivity of DLD-1 cells to PTX at different concentrations of PTX and PZQ. Colony formation assay also showed that the combination of PZQ and PTX remarkably suppressed colony formation compared with either agent treatment alone (Fig. 1C). Median Dose Effect analysis was used to characterize interactions between PZQ and PTX in regard to decline in cell viability. Combination index values <1.0, derived from the effect of a range of PZQ and PTX concentrations in DLD-1 and H1299 cell lines, indicated a synergistic effect between PZQ and PTX (Fig. 1D).

### Effect of the Combination of PZQ and PTX on Apoptosis Induction

**Figure 2:**
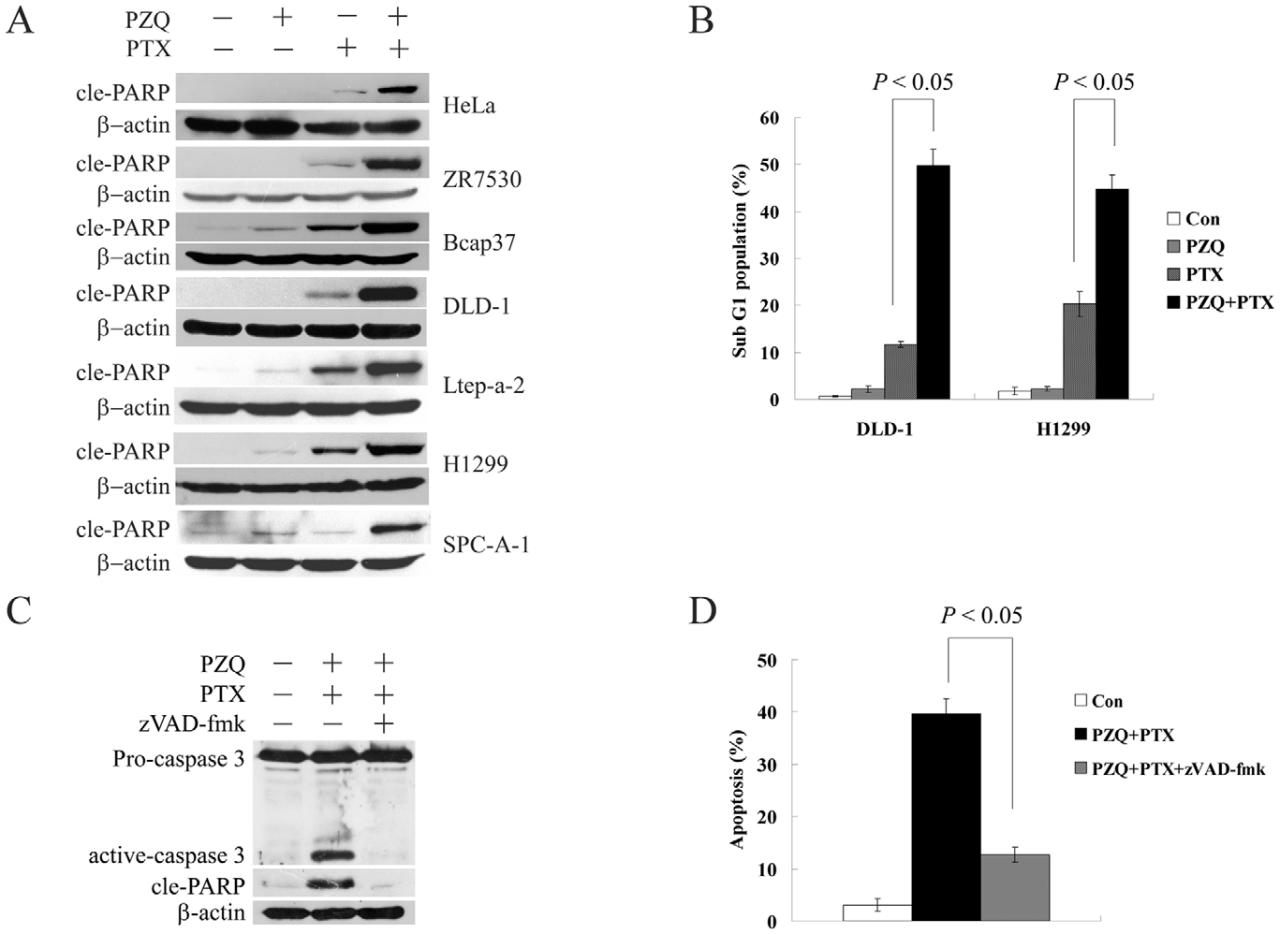
PZQ enhances PTX-induced apoptosis in various cancer cell lines. (A) The indicated cell lines were treated as in Fig. 1A, and apoptosis was examined by Western analysis of the cleaved PARP (cle-PARP) level. (B) DLD-1 and H1299 cells were treated with 20 µM PZQ alone, 10 nM PTX alone, or the combination for 48 h. Then sub-G1 fraction was determined by flow cytometry. (C) DLD-1 cells were treated with 20 µM PZQ and 10 nM PTX in the absence or presence of 20 µM caspase inhibitor zVAD-fmk for 48 h, and the expression of caspase 3 and cle-PARP were examined by Western blot. (D) After DLD-1 cells were treated as in C, the percentage of apoptotic cells was determined by DAPI staining. All Values represent mean±SEM from three separate experiments.

To further confirm potentiation of PTX-induced cell death by PZQ, we analyzed cell extracts for expression of apoptotic markers such as poly (ADP-ribose) polymerase (PARP) cleavage. The combination resulted in marked cleavage of PARP in a variety of different cell lines, whereas administration of PZQ or PTX alone did not (Fig. 2A), indicating significant activation of the apoptotic cascade by this combination. Moreover, the percentage of sub-G1 population was determined by flow cytometry. As shown in Fig. 2B, compared to treatment with either agent alone, the co-treatment of PZQ with PTX markedly increased the accumulation of cells in the sub-G1 phase. We also confirmed these results by nuclear staining with DAPI, an alternative cell apoptosis assay. The percentage of cells with apoptotic nuclei was significantly higher in the combination group than in the single-treatment group (data not shown). We next examined the contribution of caspases to apoptosis induced by the co-treatment of PZQ and PTX. As shown in Fig. 2C, caspase 3 activation and PARP cleavage were blocked by the broad-spectrum caspase inhibitor zVAD-fmk. Treatment with zVAD-fmk also greatly blocked apoptosis induced by the co-treatment of PZQ and PTX (Fig. 2D). These results suggested that cell apoptosis induced by the co-treatment of PTX and PZQ depended on caspase activity.

### The Co-treatment of PZQ and PTX could Induce Mitotic Arrest in Tumor Cells

**Figure 3:**
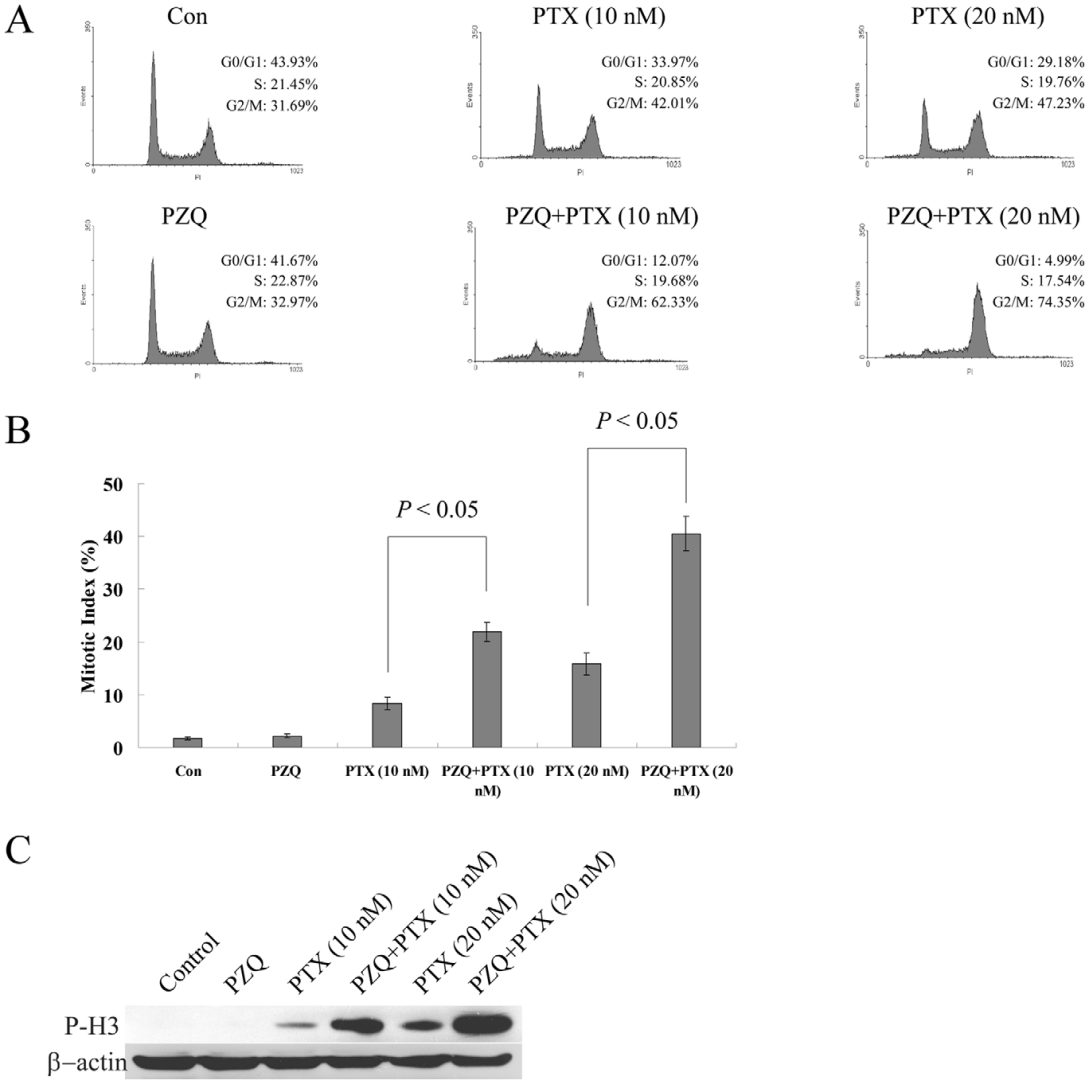
The co-treatment of PZQ and PTX could induce mitotic arrest. (A) DLD-1 cells were treated with 10 nM or 20 nM PTX in the absence or presence of 20 µM PZQ for 12 h, and cell cycle was analyzed by flow cytometry. The results are representative of three independent experiments. After DLD-1 cells were treated as above, mitotic index was determined as described in “Materials and Methods” (B), and expression level of phospho-histone H3 (Ser-10) (P-H3) was monitored by Western blot (C). Values represent mean±SEM from three separate experiments.

Flow cytometry was used to examine the effects of PZQ and PTX on the cell cycle distribution of tumor cells. The co-treatment of PZQ and PTX dose-dependently increased the G2/M population compared with PTX treatment alone in DLD-1 cells (Fig. 3A). To clarify the profile of G2/M-accumulated cells induced by the combination, we examined mitotic index in DLD-1 cells treated with PTX in the absence or presence of PZQ. As expected, PTX treatment alone dose-dependently increased mitotic index (Fig. 3B). PTX in combination with PZQ further led to an increase in mitotic index (Fig. 3B). We also found that PZQ could dramatically promote PTX-mediated increase in the expression level of phosphorylated histone H3 (Ser-10) (P-H3), a mitotic marker (Fig. 3C). These results suggested that PZQ enhanced PTX-induced mitotic arrest in tumor cells.

**Figure 4:**
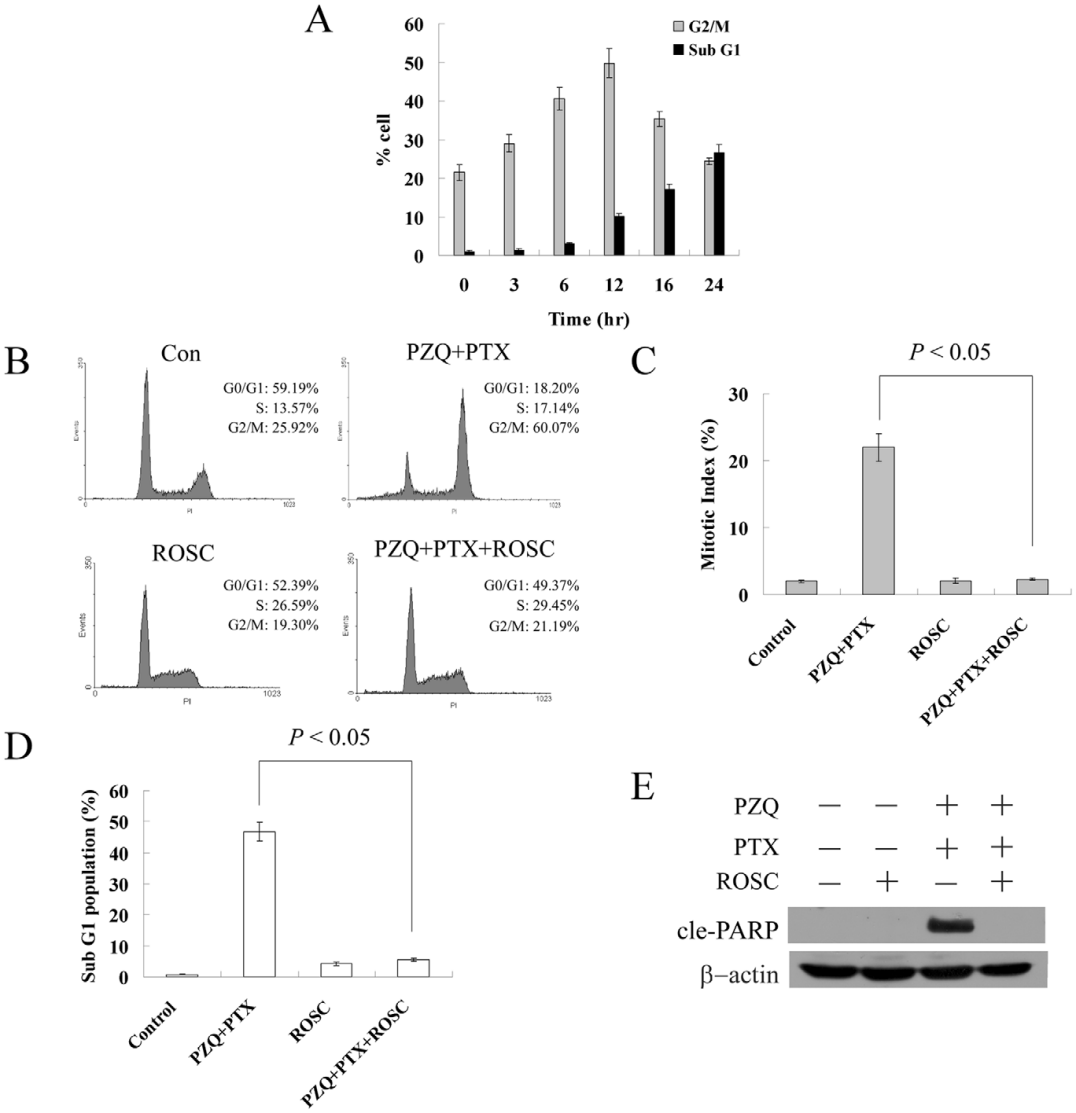
Apoptosis induced by the co-treatment of PZQ and PTX may result from the mitotic arrest induced by this treatment. (A) After DLD-1 cells were co-treated with 20 µM PZQ and 10 nM PTX for indicated time points, the G2/M and Sub-G1 fractions were determined by flow cytometry. (B) DLD-1 cells were treated with a combination of PZQ (20 µM) and PTX (10 nM) with or without 12.5 µM roscovitine for 12 h, and then cell cycle was analyzed by flow cytometry. (C) DLD-1 cells were treated as in (B), and mitotic index was determined. After DLD-1 were treated with a combination of PZQ (20 µM) and PTX (10 nM) in the presence or absence of 12.5 µM roscovitine for 48 h, Apoptosis was assessed by flow cytometry analysis of Sub-G1 population (D) and Western analysis of cle-PARP level (E). All Values are shown as mean±SEM from three separate experiments.

A time course experiment based on flow cytometry showed that the increase in the G2/M population preceded that of the sub-G1 population in DLD-1 cells treated with the combination of PZQ and PTX (Fig. 4A), indicating persistent G2/M arrest probably led to apoptosis. We then examined whether there was a correlation between mitotic arrest and apoptosis induced by the co-treatment of PZQ and PTX. An inhibitor of CDK, roscovitine, was used. Roscovitine could selectively inhibit CDK1, CDK2 and CDK5 and block cell cycle progression by preventing cells from entering the S and M phases [32]. As shown in Fig. 4B, roscovitine almost completely inhibited the G2/M arrest induced by the co-treatment of PZQ and PTX in DLD-1 cells. The increase in the mitotic index resulted from the co-treatment of PZQ and PTX also returned to the control level after addition of roscovitine (Fig. 4C), indicating that roscovitine inhibited the mitotic arrest induced by this combination. Strikingly, roscovitine also inhibited PZQ-enhanced PTX-mediated apoptosis almost completely, as determined by apoptotic sub-G1 population and Western analysis of cleaved PARP (Fig. 4D and E). Similar results were obtained with H1299 cells (data not shown). These data suggested that increased apoptosis induced by the co-treatment of PZQ and PTX likely resulted from increased mitotic arrest induced by this co-treatment.

### The Co-treatment of PZQ and PTX could Down-regulate XIAP Protein

**Figure 5:**
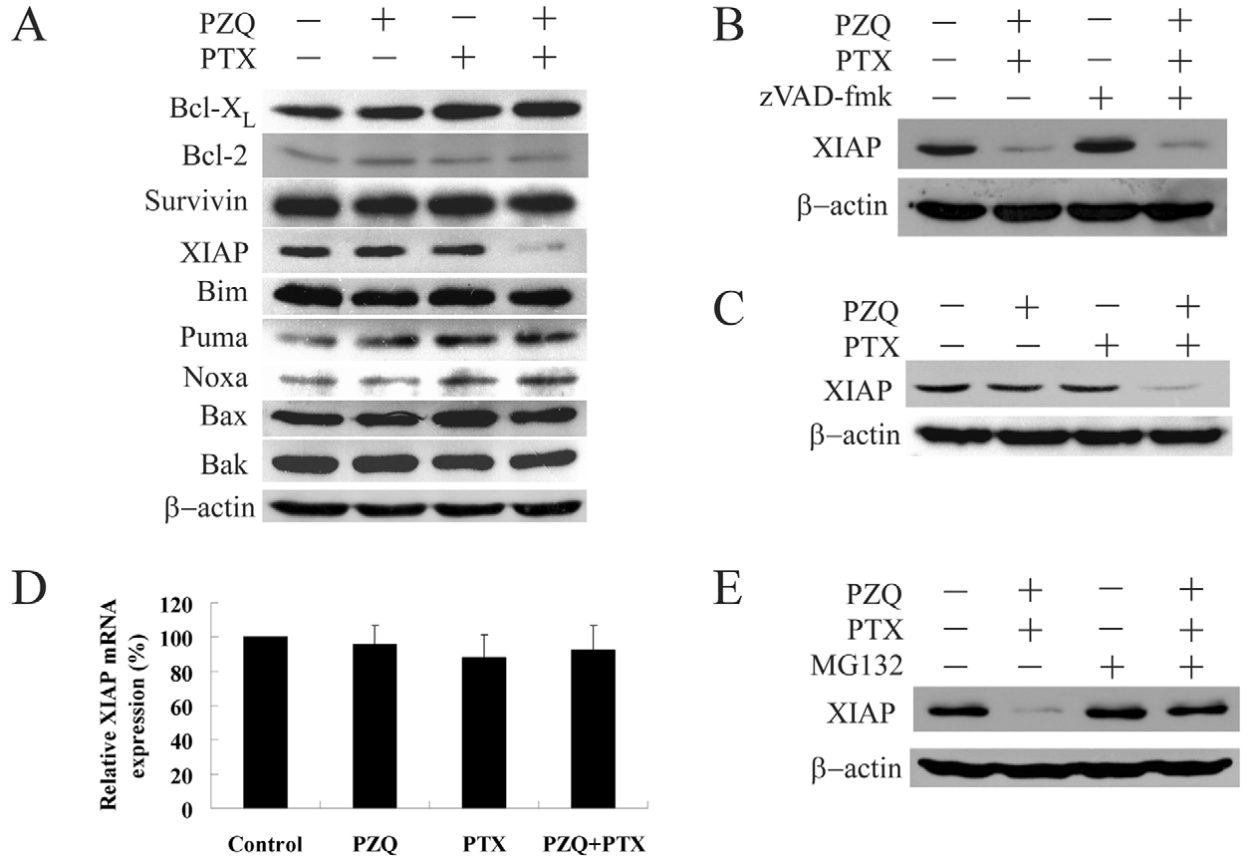
The co-treatment of PZQ and PTX could suppress XIAP protein. (A) DLD-1 cells were treated with 20 µM PZQ alone, 10 nM PTX alone, or the combination for 24 h. Then expression of Bcl-XL, Bcl-2, Survivin, XIAP, Bim, Puma, Noxa, Bax and Bak were monitored by Western blot. (B) After exposure of DLD-1 cells to a combination of 20 µM PZQ and 10 nM PTX in the presence or absence of 20 µM zVAD-fmk for 24 h, expression of XIAP was determined by Western blot. (C) H1299 cells were treated with 20 µM PZQ alone, 10 nM PTX alone, or the combination for 24 h, after which expression of XIAP was examined. (D) After DLD-1 cells were treated as in (A), total RNA was isolated and XIAP mRNA was quantified by real-time RT-PCR. Following normalization to GAPDH mRNA, the XIAP mRNA expression values for each condition were relative to vehicle-treated control cells. (E) DLD-1 cells were treated with a combination of 20 µM PZQ and 10 nM PTX in the absence or presence of 10 µM MG132 for 24 h, and then expression of XIAP was monitored by Western blot. Values represent mean±SEM from three separate experiments.

To evaluate the underlying mechanism of synergistic cytotoxicity between PZQ and PTX, we assessed the effects of the two agents, either alone or in combination, on various apoptosis regulatory proteins in DLD-1 cells. As shown in Fig. 5A, treatment with PZQ or PTX alone did not alter expression of XIAP. However, exposure of cells to the combination of PZQ and PTX resulted in a remarkable decrease in level of XIAP. No significant changes in the expression of other proteins, such as Bcl-X_L_, surviving, Puma, Bim, Noxa, Bax and Bak, were observed in cells exposed to PTX alone, PZQ alone, or the combination.

To determine whether changes in expression of XIAP were dependent on caspase activation, we treated DLD-1 cells with the drug combination in the presence or absence of the caspase inhibitor zVAD-fmk. As shown in Fig. 5B, zVAD-fmk had little effect on down-regulation of XIAP induced by the combination of PZQ and PTX, indicating that changes in expression of XIAP were caspase-independent. Down-regulation of XIAP was also observed in H1299 cells co-treated with PZQ and PTX (Fig. 5C), suggesting that modulation of this protein may not be cell line specific.

We tried to identify the mechanism by which XIAP was down-regulated in response to the co-treatment of PZQ and PTX. To investigate whether the co-treatment of PZQ and PTX could regulate XIAP expression at the transcriptional level, we used reverse transcription-PCR. Compared with the control group, combined treatment with PZQ and PTX did not lead to significant change in the mRNA levels of XIAP (Fig. 5D).

Another possible explanation for XIAP down-regulation could be degradation of XIAP by the proteasome. To test this hypothesis, we treated DLD-1 cells with the drug combination in the presence of a proteasome inhibitor, MG132. Suppression of XIAP in DLD-1 cells treated with the combination of PZQ and PTX was almost completely abolished by MG132 (Fig. 5E). These results indicated that PZQ and PTX might induce XIAP degradation via the proteasome-mediated pathway.

### XIAP Plays an Important Role in the Synergistic Cytotoxicity of PZQ and PTX

**Figure 6:**
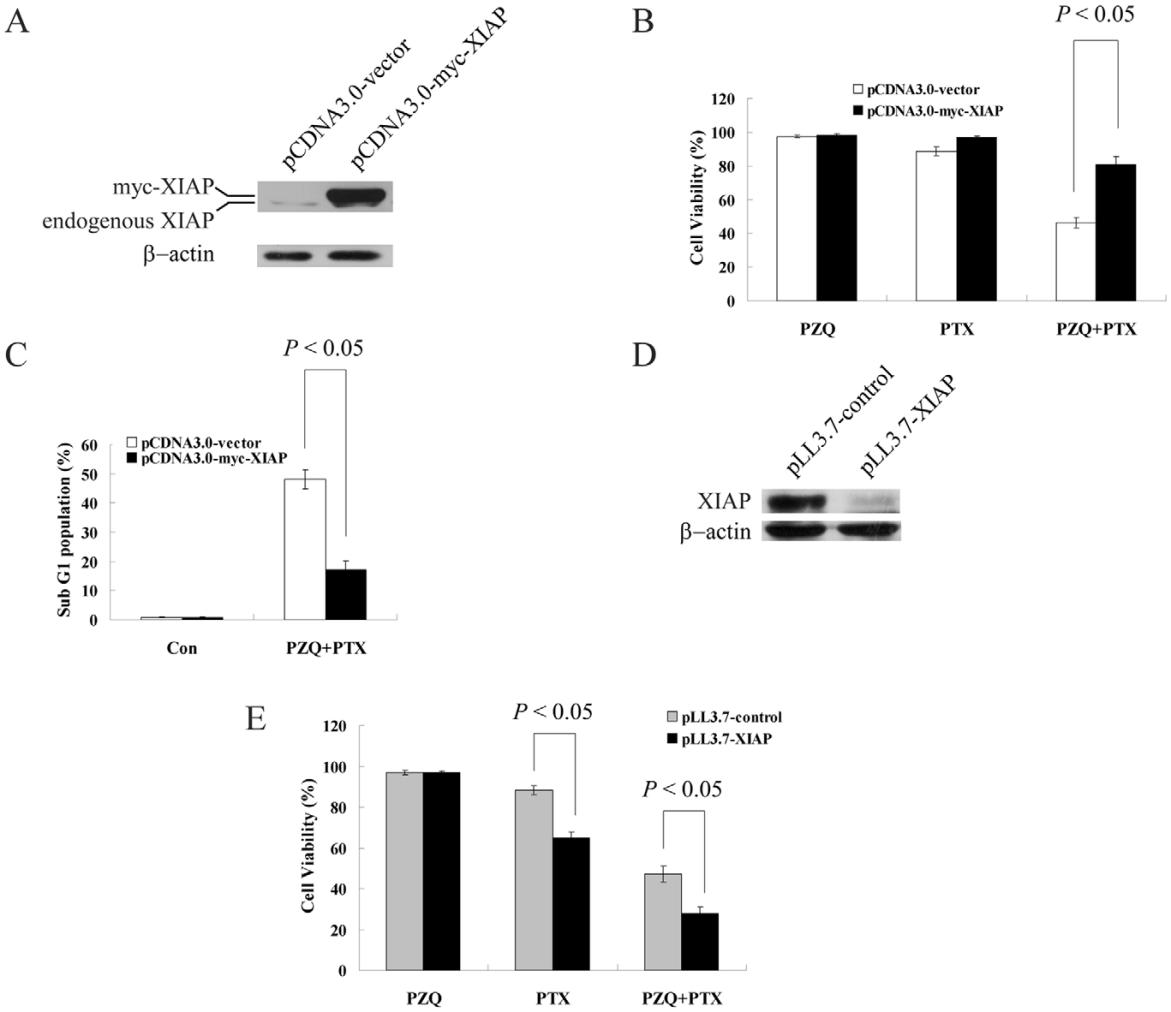
XIAP plays an important role in the synergistic cytotoxicity of PZQ and PTX. (A) Western blot showed XIAP expression in DLD-1 cells at 24 h after transfection with pcDNA3-myc-XIAP or control vector. (B) 24 h after transfection with pcDNA3-myc-XIAP and control vector, DLD-1 cells were treated with 20 µM PZQ alone, 10 nM PTX alone or the combination for 48 h, and then cell viability was determined by MTT assay. (C) 24 h after transfection with pcDNA3-myc-XIAP and control vector, DLD-1 cells were treated with a combination of 20 µM PZQ and 10 nM PTX. Then the sub-G1 fraction was determined by flow cytometry. (D) DLD-1 cells were infected with a lentivirus encoding control shRNA or XIAP shRNA for 48 h, and expression of XIAP was analyzed by Western blot. (E) DLD-1 cells were infected with a lentivirus encoding control shRNA or XIAP shRNA for 48 h, and then treated with 20 µM PZQ alone, 10 nM PTX alone or the combination for 48 h. The cell viability was determined by MTT assay. All Values are shown as mean±SEM from three separate experiments.

Based on the above results,we investigated whether downregulation of XIAP actually mediated cell death induced by the combination of PZQ and PTX. We transiently transfected DLD-1 cells with a plasmid containing epitope-tagged XIAP (myc-XIAP) and an empty plasmid that served as a control. Transfection was confirmed by Western blot (Fig. 6A). Overexpression of XIAP in DLD-1 cells significantly attenuated PZQ-enhanced PTX-induced cytotoxicity, as determined by analysis of cell viability and sub-G1 population (Fig. 6B and C).

To further study the role of XIAP in the synergy between PZQ and PTX, we used lentivirus-mediated short hairpin RNA (shRNA) to knockdown XIAP in DLD-1 cells. Cells were harvested at 48 h after infection, and XIAP expression was analyzed by Western blot. Compared with DLD-1 cells infected with the control shRNA, cells infected with XIAP shRNA displayed dramatically suppressed XIAP expression (Fig. 6D). We next assessed the effect of XIAP knockdown on cell viability. As shown in Fig. 6E, blocking expression of XIAP had no effect on the sensitivity of DLD-1 cells to PZQ treatment. However, XIAP knockdown significantly sensitized DLD-1 cells to PTX-induced cytotoxicity (Fig. 6E). Moreover, cytotoxicity induced by the co-treatment of PZQ and PTX was also enhanced in XIAP-knockdown DLD-1 cells (Fig. 6E). These data suggest that XIAP plays a significant role in mediating the synergistic cytotoxicity caused by the co-treatment of PZQ and PTX.

### The Combination of PZQ and PTX Suppresses Tumor Growth *in vivo*

**Figure 7:**
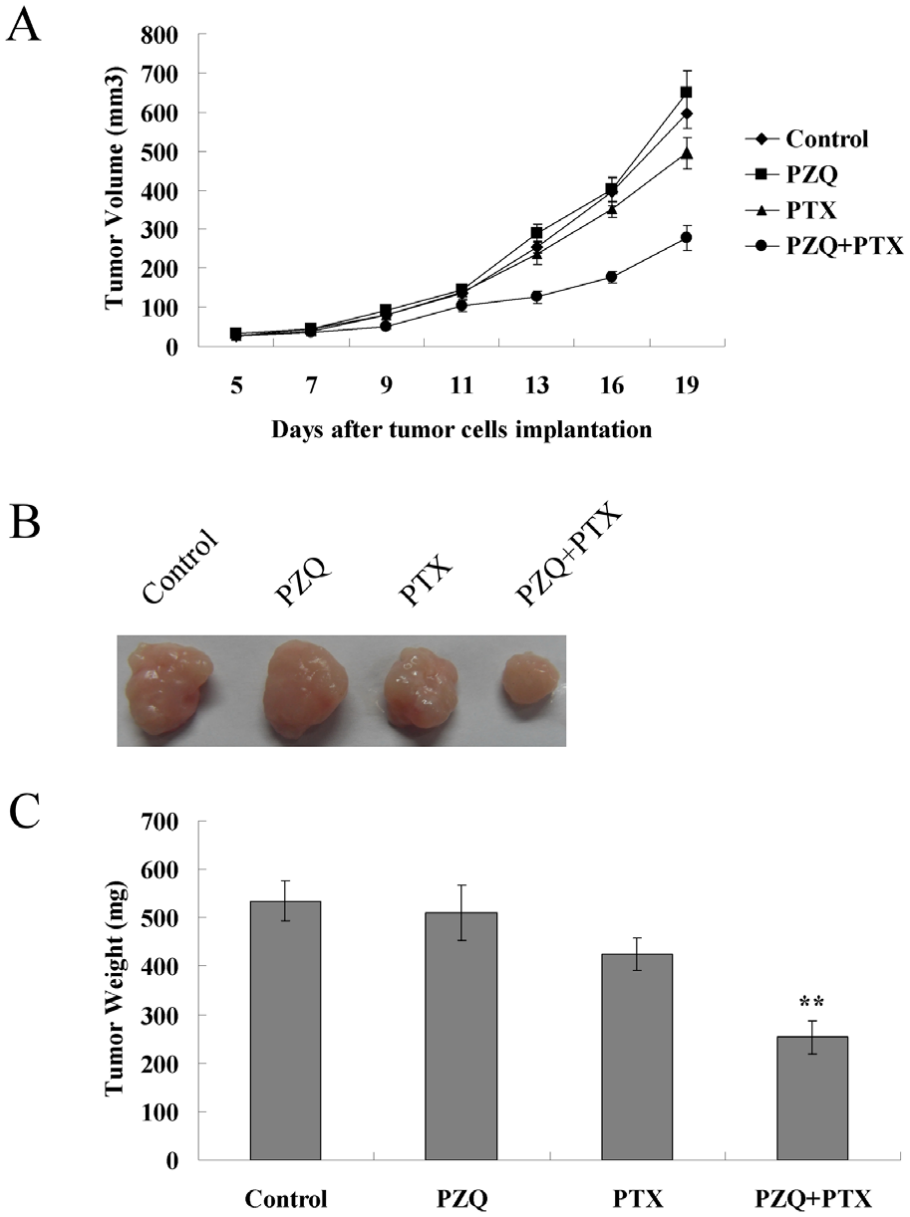
Potentiation of PTX-induced cytotoxicity by PZQ *in vivo*. (A) Athymic nude mice were received subcutaneous injections with DLD-1 cells (2×10^6^), and grown for 5 days (tumor volume reached 25–35 mm^3^) before initiation of treatment. These mice (n = 6 per group) were injected intraperitoneally with vehicle, PZQ alone (50 mg/kg), PTX alone (30 mg/kg), or combined both. PZQ was given on days 5, 7, 9, 11, and 13, and PTX was given on days 5, 9, and 13 after injection of DLD-1 cells. The tumor volumes were measured on the indicated days. (B) On day 19, xenograft tumor from each group were removed and photographed. Representative tumor in each group was shown. (C) After xenograft tumors were removed, these tumors were weighted. Data are shown as mean±SEM (*n* = 6 mice, each group). **, p<0.05, compared with single agent-treated groups and the vehicle-treated (control) group.

To test if the co-treatment of PZQ and PTX has any effect on tumor growth *in vivo*, we established a xenograft model in nude mice with PTX-resistant DLD-1 cells. Subcutaneously injected DLD-1 cells gave rise to exponentially growing tumors in athymic nude mice (Fig. 7A). Combined treatment with PZQ and PTX greatly suppressed tumor growth relative to PTX treatment alone, whereas PZQ administered alone was unable to inhibit tumor growth in the DLD-1 xenograft–bearing mice (Fig. 7A and B). Compared to the control group, the treatment with PZQ and PTX combination resulted in a >50% reduction in tumor weight (Fig. 7C). Weight loss was not noted throughout the different treatment cycles, and no mice died during the observation period.

## Discussion

PZQ has gained considerable interest as an antiparasitic drug with no obvious side effect. In this study, we showed the anticancer potential of PZQ for the first time. PZQ could potentiate the growth-inhibitory effect of PTX in various tumor cells, including those resistant to PTX. The co-treatment of PZQ and PTX could also dramatically activate the apoptosis cascade, accompanied by perturbations in survival and signaling regulatory proteins. The synergistic anticancer effect of PZQ and PTX was further confirmed in a mouse xenograft model. Because PZQ is a clinically used drug, our findings may be readily translated into clinical practice and have important implications for PTX-based therapy especially in dealing with PTX-resistant cases.

We found that PZQ itself did not exert any effect on cell cycle. However, PZQ could greatly enhance PTX-induced G2/M arrest in DLD-1 and H1299 cells, and cell cycle arrest occurred at the mitosis phase, which was reflected by elevated mitotic index and P-H3 protein level. Microtubule-stabilizing agents like PTX can bind directly to tubulin and stabilize microtubule, which disrupts the dynamic assembly of the mitotic spindle and leads to mitotic arrest [21], [22]. Numerous studies have demonstrated the importance of mitotic arrest in PTX-induced apoptosis [33], [34]. Our time course analysis of cell cycle also suggested that the G2/M arrest preceded the apoptosis induction when DLD-1 cells were treated with the combination of PZQ and PTX. Furthermore, inhibition of mitotic arrest by the CDK inhibitor roscovitine almost completely blocked apoptosis induced by this combination. These results revealed that the mitotic arrest caused by the co-treatment of PZQ and PTX played an important role in following apoptosis induction.

The specific role of the anti-apoptotic protein XIAP in interactions between PZQ and PTX was investigated in our study, because we found that XIAP expression was strongly reduced in DLD-1 and H1299 cells on treatment with this combination and inhibition of XIAP occurred upstream of caspase activation. XIAP, a potent endogenous inhibitor of caspase, is frequently overexpressed in many tumors and, in certain cancer type, is correlated to tumor progression [35], [36]. Inhibition of XIAP could reduce tumorigenicity and enhance therapeutic sensitivity of cancer cells [36], [37]. Thus, XIAP has been an attractive target for the treatment of malignancy. Here we showed that overexpression of XIAP could protect cells from apoptosis induced by the co-treatment of PZQ and PTX. Although XIAP overexpression can protect cells against apoptosis induced by multiple agents, the relevance of XIAP down-regulation to the synergism between PZQ and PTX was supported by our subsequent studies using lentivirus-mediated knockdown of XIAP. Knockdown of XIAP significantly increased cell sensitivity to PTX and its combination with PZQ. These results supported the fact that suppression of XIAP was a key mechanism for apoptosis induced by the co-treatment of PTX and PZQ. We also identified the mechanisms through which XIAP was down-regulated in DLD-1 cells on treatment with the PZQ and PTX co-treatment. Although the mRNA level of XIAP was not changed by the co-treatment of PTX and PZQ, the proteasome inhibitor MG132 could greatly inhibit the down-regulation of XIAP induced by this co-treatment, suggesting that the co-treatment of PTX and PZQ may promote activation of the proteasome pathway to degrade XIAP. XIAP has ubiquitin ligase activity. The self-ubiquitination and degradation of XIAP is an important mechanism for its stability and function regulation [36], [38]. Further study will be required to elucidate the mechanism of the synergistic effect on XIAP by PZQ and PTX.

Taken together, we showed that the co-treatment of PZQ and PTX could exert synergistic cytotoxicity to inhibit cancer cell growth both *in vitro* and *in vivo*. Although the mechanism of this synergistic interaction was not fully understood yet, we did get some insights into the mechanism. The current data support that PZQ in combination with PTX could be a new therapeutic regimen in dealing with PTX-resistant tumor. Such a combination of two already clinically used drugs could also reduce clinical safety concerns while performing better anti-cancer efficacy.

## Supporting Information

Figure S1 **PZQ does not exert cytotoxicity on tumor cells.** (A) HeLa, ZR7530, Bcap37, DLD-1, Ltep-a-2, H1299 and SPC-A-1 cells were treated with 50 µM or 100 µM PZQ for 48 h. Then cell viability was determined by MTT assay. (B) DLD-1 and H1299 cells were cultured in the absence or presence of 100 µM PZQ for 10 days, and then colonies were stained with crystal violet. (C) DLD-1 and H1299 cells were treated with or without 100 µM PZQ for 48 h and then stained with DAPI. Bars = 10 µm. (D) After DLD-1 and H1299 cells were treated with 50 µM or 100 µM PZQ for 48 h, the Sub-G1 population was analyzed by flow cytometry.
(TIF)

Figure S2 **Cytotoxicity profile of PTX in a panel of tumor cell lines.** Cells were incubated with PTX for 48 h, and cell viability was measured by MTT assay. IC50 values were determined by curve analysis software (GraphPad Prism). Data are mean±SEM of 3 independent experiments. DLD-1 and H1299 cell lines show significant resistance to PTX.
(TIF)

## References

[1] Chong CR, Sullivan DJ Jr (2007) New uses for old drugs. Nature 448: 645–646. 17687303 10.1038/448645a

[2] Boguski MS, Mandl KD, Sukhatme VP (2009) Drug discovery. Repurposing with a difference. Science 324: 1394–1395. 19520944 10.1126/science.1169920

[3] Barlogie B, Tricot G, Anaissie E, Shaughnessy J, Rasmussen E, et al (2006) Thalidomide and hematopoietic-cell transplantation for multiple myeloma. N Engl J Med 354: 1021–1030. 16525139 10.1056/NEJMoa053583

[4] Chen Q, Espey MG, Sun AY, Pooput C, Kirk KL, et al (2008) Pharmacologic doses of ascorbate act as a prooxidant and decrease growth of aggressive tumor xenografts in mice. Proc Natl Acad Sci U S A 105: 11105–11109. 18678913 10.1073/pnas.0804226105 PMC2516281

[5] Ichim TE, Minev B, Braciak T, Luna B, Hunninghake R, et al (2011) Intravenous ascorbic acid to prevent and treat cancer-associated sepsis? J Transl Med 9: 25–37. 21375761 10.1186/1479-5876-9-25 PMC3061919

[6] Fromberg A, Gutsch D, Schulze D, Vollbracht C, Weiss G, et al (2011) Ascorbate exerts anti-proliferative effects through cell cycle inhibition and sensitizes tumor cells towards cytostatic drugs. Cancer Chemother Pharmacol 67: 1157–1166. 20694726 10.1007/s00280-010-1418-6 PMC3082037

[7] Flossmann E, Rothwell PM (2007) Effect of aspirin on long-term risk of colorectal cancer: consistent evidence from randomised and observational studies. Lancet 369: 1603–1613. 17499602 10.1016/S0140-6736(07)60747-8

[8] Rothwell PM, Wilson M, Elwin CE, Norrving B, Algra A, et al (2010) Long-term effect of aspirin on colorectal cancer incidence and mortality: 20-year follow-up of five randomised trials. Lancet 376: 1741–1750. 20970847 10.1016/S0140-6736(10)61543-7

[9] Zhou H, Liu W, Su Y, Wei Z, Liu J, et al (2010) NSAID sulindac and its analog bind RXRalpha and inhibit RXRalpha-dependent AKT signaling. Cancer Cell 17: 560–573. 20541701 10.1016/j.ccr.2010.04.023 PMC2907921

[10] Grosch S, Maier TJ, Schiffmann S, Geisslinger G (2006) Cyclooxygenase-2 (COX-2)-independent anticarcinogenic effects of selective COX-2 inhibitors. J Natl Cancer Inst 98: 736–747. 16757698 10.1093/jnci/djj206

[11] Haanen C (2001) Sulindac and its derivatives: a novel class of anticancer agents. Curr Opin Investig Drugs 2: 677–683. 11569947

[12] Woerdenbag HJ, Moskal TA, Pras N, Malingre TM, el-Feraly FS, et al (1993) Cytotoxicity of artemisinin-related endoperoxides to Ehrlich ascites tumor cells. J Nat Prod 56: 849–856. 8350087 10.1021/np50096a007

[13] Efferth T, Dunstan H, Sauerbrey A, Miyachi H, Chitambar CR (2001) The anti-malarial artesunate is also active against cancer. Int J Oncol 18: 767–773. 11251172 10.3892/ijo.18.4.767

[14] Hou J, Wang D, Zhang R, Wang H (2008) Experimental therapy of hepatoma with artemisinin and its derivatives: in vitro and in vivo activity, chemosensitization, and mechanisms of action. Clin Cancer Res 14: 5519–5530. 18765544 10.1158/1078-0432.CCR-08-0197

[15] Nakase I, Lai H, Singh NP, Sasaki T (2008) Anticancer properties of artemisinin derivatives and their targeted delivery by transferrin conjugation. Int J Pharm 354: 28–33. 17942255 10.1016/j.ijpharm.2007.09.003

[16] Posner GH, Paik IH, Sur S, McRiner AJ, Borstnik K, et al (2003) Orally active, antimalarial, anticancer, artemisinin-derived trioxane dimers with high stability and efficacy. J Med Chem 46: 1060–1065. 12620083 10.1021/jm020461q

[17] Gonnert R, Andrews P (1977) Praziquantel, a new board-spectrum antischistosomal agent. Z Parasitenkd 52: 129–150. 410178 10.1007/BF00389899

[18] Keiser J, Utzinger J (2007) Food-borne trematodiasis: current chemotherapy and advances with artemisinins and synthetic trioxolanes. Trends Parasitol 23: 555–562. 17950667 10.1016/j.pt.2007.07.012

[19] Brindley PJ, Sher A (1987) The chemotherapeutic effect of praziquantel against Schistosoma mansoni is dependent on host antibody response. J Immunol 139: 215–220. 3108397

[20] Joseph S, Jones FM, Walter K, Fulford AJ, Kimani G, et al (2004) Increases in human T helper 2 cytokine responses to Schistosoma mansoni worm and worm-tegument antigens are induced by treatment with praziquantel. J Infect Dis 190: 835–842. 15272413 10.1086/422604

[21] Jordan MA, Toso RJ, Thrower D, Wilson L (1993) Mechanism of mitotic block and inhibition of cell proliferation by taxol at low concentrations. Proc Natl Acad Sci U S A 90: 9552–9556. 8105478 10.1073/pnas.90.20.9552 PMC47607

[22] Milross CG, Mason KA, Hunter NR, Chung WK, Peters LJ, et al (1996) Relationship of mitotic arrest and apoptosis to antitumor effect of paclitaxel. J Natl Cancer Inst 88: 1308–1314. 8797771 10.1093/jnci/88.18.1308

[23] Jordan MA, Wilson L (2004) Microtubules as a target for anticancer drugs. Nat Rev Cancer 4: 253–265. 15057285 10.1038/nrc1317

[24] Rodriguez-Antona C (2010) Pharmacogenomics of paclitaxel. Pharmacogenomics 11: 621–623. 20415548 10.2217/pgs.10.32

[25] Gottesman MM (2002) Mechanisms of cancer drug resistance. Annu Rev Med 53: 615–627. 11818492 10.1146/annurev.med.53.082901.103929

[26] Aoki D, Oda Y, Hattori S, Taguchi K, Ohishi Y, et al (2009) Overexpression of class III beta-tubulin predicts good response to taxane-based chemotherapy in ovarian clear cell adenocarcinoma. Clin Cancer Res 15: 1473–1480. 19228748 10.1158/1078-0432.CCR-08-1274

[27] Qi Y, Fu X, Xiong Z, Zhang H, Hill SM, et al (2012) Methylseleninic acid enhances paclitaxel efficacy for the treatment of triple-negative breast cancer. PLoS One 7: e31539. 22348099 10.1371/journal.pone.0031539 PMC3279411

[28] Le XF, Mao W, He G, Claret FX, Xia W, et al (2011) The role of p27(Kip1) in dasatinib-enhanced paclitaxel cytotoxicity in human ovarian cancer cells. J Natl Cancer Inst 103: 1403–1422. 21813412 10.1093/jnci/djr280 PMC3176777

[29] Harasawa R, Mizusawa H, Nozawa K, Nakagawa T, Asada K, et al (1993) Detection and tentative identification of dominant mycoplasma species in cell cultures by restriction analysis of the 16S–23S rRNA intergenic spacer regions. Res Microbiol 144: 489–493. 7910696 10.1016/0923-2508(93)90057-9

[30] Tang J, Hu M, Lee S, Roblin R (1999) Primer mixture enhances PCR detection of Mycoplasma/Acholeplasma contaminants in cell cultures. In Vitro Cell Dev Biol Anim 35: 1–3. 10475245 10.1007/s11626-999-0033-5

[31] Wu ZH, Hu LY, Xu DQ, Li X (2012) A cell-based assay for screening spindle checkpoint inhibitors. Assay Drug Dev Technol 10: 344–352. 22352901 10.1089/adt.2011.416 PMC3421965

[32] Meijer L, Borgne A, Mulner O, Chong JP, Blow JJ, et al (1997) Biochemical and cellular effects of roscovitine, a potent and selective inhibitor of the cyclin-dependent kinases cdc2, cdk2 and cdk5. Eur J Biochem 243: 527–536. 9030781 10.1111/j.1432-1033.1997.t01-2-00527.x

[33] Swanton C, Marani M, Pardo O, Warne PH, Kelly G, et al (2007) Regulators of mitotic arrest and ceramide metabolism are determinants of sensitivity to paclitaxel and other chemotherapeutic drugs. Cancer Cell 11: 498–512. 17560332 10.1016/j.ccr.2007.04.011

[34] Akiyoshi T, Nakamura M, Yanai K, Nagai S, Wada J, et al (2008) Gamma-secretase inhibitors enhance taxane-induced mitotic arrest and apoptosis in colon cancer cells. Gastroenterology 134: 131–144. 18166351 10.1053/j.gastro.2007.10.008

[35] Takeuchi H, Kim J, Fujimoto A, Umetani N, Mori T, et al (2005) X-Linked inhibitor of apoptosis protein expression level in colorectal cancer is regulated by hepatocyte growth factor/C-met pathway via Akt signaling. Clin Cancer Res 11: 7621–7628. 16278380 10.1158/1078-0432.CCR-05-0479

[36] Schimmer AD, Dalili S, Batey RA, Riedl SJ (2006) Targeting XIAP for the treatment of malignancy. Cell Death Differ 13: 179–188. 16322751 10.1038/sj.cdd.4401826

[37] OConnor CL, Anguissola S, Huber HJ, Dussmann H, Prehn JH, et al (2008) Intracellular signaling dynamics during apoptosis execution in the presence or absence of X-linked-inhibitor-of-apoptosis-protein. Biochim Biophys Acta 1783: 1903–1913. 18590777 10.1016/j.bbamcr.2008.05.025

[38] Yang Y, Fang S, Jensen JP, Weissman AM, Ashwell JD (2000) Ubiquitin protein ligase activity of IAPs and their degradation in proteasomes in response to apoptotic stimuli. Science 288: 874–877. 10797013 10.1126/science.288.5467.874

