# Supplementary figures and images for: Praziquantel Synergistically Enhances Paclitaxel Efficacy to Inhibit Cancer Cell Growth
Source: PLoS One. 2012 Dec 12;7(12):e51721. doi: 10.1371/journal.pone.0051721 (PMC3520897; doi:10.1371/journal.pone.0051721)

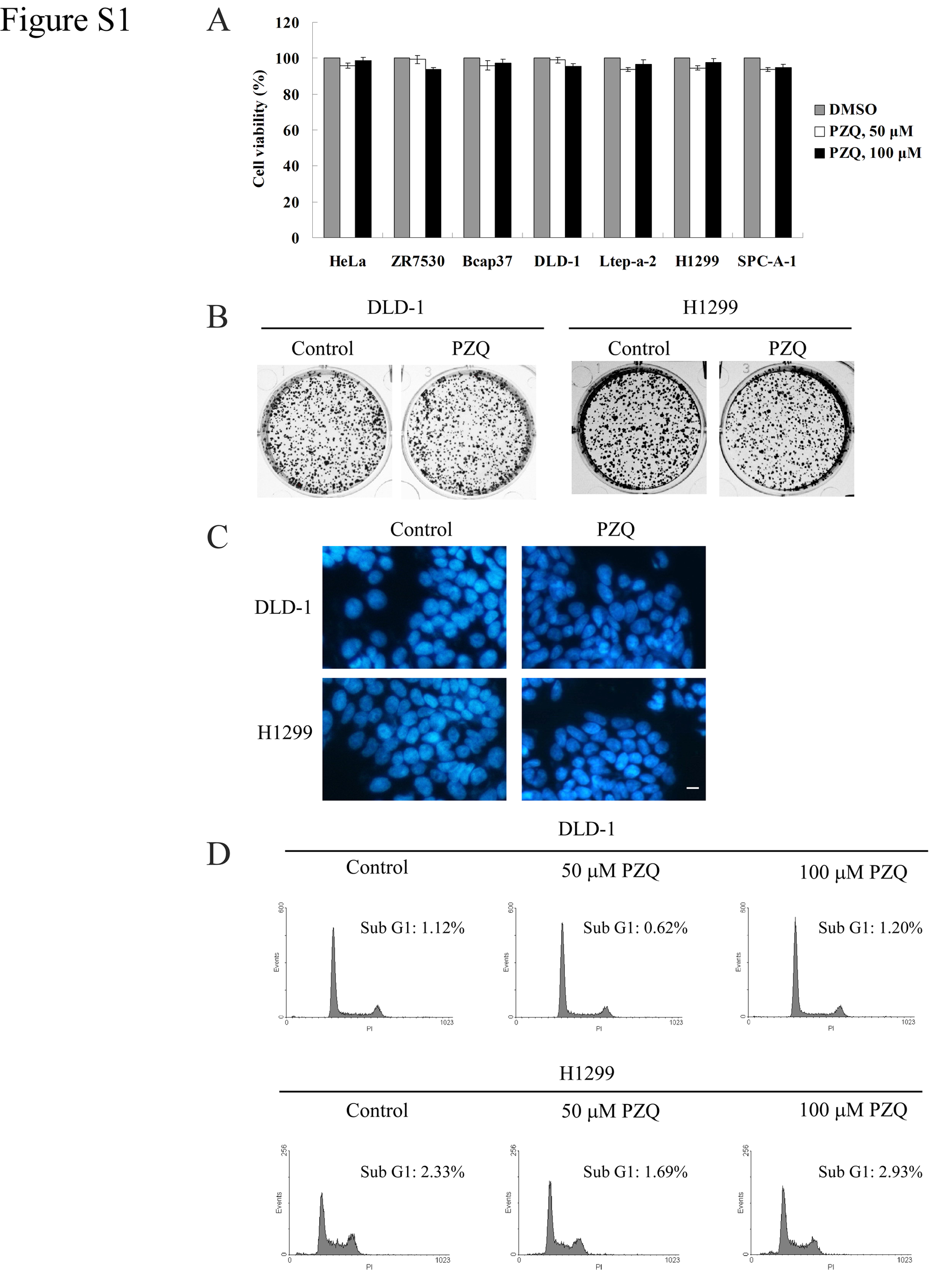

Supplement: Figure S1 — PZQ does not exert cytotoxicity on tumor cells. (A) HeLa, ZR7530, Bcap37, DLD-1, Ltep-a-2, H1299 and SPC-A-1 cells were treated with 50 µM or 100 µM PZQ for 48 h. Then cell viability was determined by MTT assay. (B) DLD-1 and H1299 cells were cultured in the absence or presence of 100 µM PZQ for 10 days, and then colonies were stained with crystal violet. (C) DLD-1 and H1299 cells were treated with or without 100 µM PZQ for 48 h and then stained with DAPI. Bars = 10 µm. (D) After DLD-1 and H1299 cells were treated with 50 µM or 100 µM PZQ for 48 h, the Sub-G1 population was analyzed by flow cytometry. (TIF) [file pone.0051721.s001.tif]

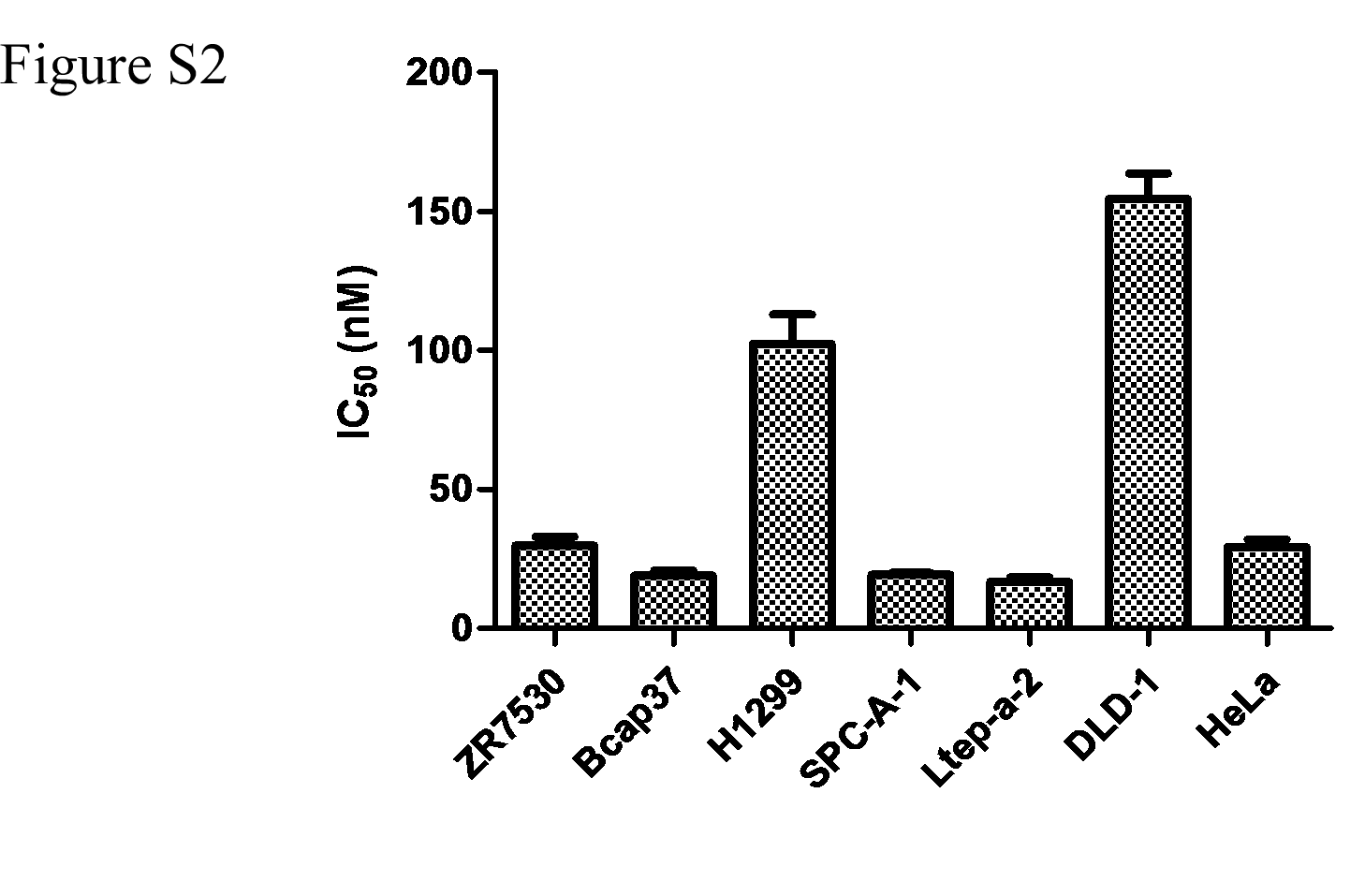

Supplement: Figure S2 — Cytotoxicity profile of PTX in a panel of tumor cell lines. Cells were incubated with PTX for 48 h, and cell viability was measured by MTT assay. IC50 values were determined by curve analysis software (GraphPad Prism). Data are mean±SEM of 3 independent experiments. DLD-1 and H1299 cell lines show significant resistance to PTX. (TIF) [file pone.0051721.s002.tif]
